# Immunization With a Combination of Four Recombinant *Brucella abortus* Proteins Omp16, Omp19, Omp28, and L7/L12 Induces T Helper 1 Immune Response Against Virulent *B. abortus* 544 Infection in BALB/c Mice

**DOI:** 10.3389/fvets.2020.577026

**Published:** 2021-01-20

**Authors:** Tran Xuan Ngoc Huy, Trang Thi Nguyen, Alisha Wehdnesday Bernardo Reyes, Son Hai Vu, WonGi Min, Hu Jang Lee, John Hwa Lee, Suk Kim

**Affiliations:** ^1^Institute of Applied Sciences, Ho Chi Minh City University of Technology - HUTECH, Ho Chi Minh City, Vietnam; ^2^Institute of Animal Medicine, College of Veterinary Medicine, Gyeongsang National University, Jinju, South Korea; ^3^College of Veterinary Medicine, Chonbuk National University, Iksan, South Korea

**Keywords:** *Brucella abortus*, combined subunit vaccine, T helper 1 T cell, humoral immunity, macrophages

## Abstract

Protective efficiency of a combination of four recombinant *Brucella abortus* (*B. abortus*) proteins, namely outer membrane protein (Omp) 16, Omp19, Omp28, and 50S ribosomal protein L7/L12 was evaluated as a combined subunit vaccine (CSV) against *B. abortus* infection in RAW 264.7 cell line and murine model. The immunoreactivity of these four recombinant proteins as well as pCold-TF vector reacted with *Brucella*-positive serum individually, but not with *Brucella*-negative serum by immunoblotting assay. CSV-treated RAW 264.7 cells significantly induced production of IFN-γ and IL-12 while decreased IL-10 production at the late stage of infection compared to PBS-treated control cells. In addition, the enhancement of nitric oxide production together with cytokines secretion profile in CSV-treated cells proved that CSV notably activated bactericidal mechanisms in macrophages. Consistently, mice immunized with CSV strongly elicited production of pro-inflammatory cytokines TNF-α, IL-6 and MCP-1 compared to PBS control group. Moreover, the concentration of IFN-γ was >IL-10 and titers of IgG2a were also heightened compared to IgG1 in CSV-immunized mice which suggest that CSV induced predominantly T helper 1 T cell. These results suggest that the CSV used in the present study is a potential candidate as a preventive therapy against brucellosis.

## Introduction

Brucellosis remains an extremely common zoonotic disease worldwide caused by *Brucella* species that are designated as category B of potential bioterrorism agents. This potential is due to the various biological and pathogenic characteristics of *Brucella* species including being infectious via the aerosol route, being notoriously debilitating disease, having no safe and effective available vaccine for humans as well as requiring prolonged antibiotic treatment and having relapse rates of 5–10% after successful treatment (1, 2). In addition, *Brucella* is an intracellular pathogen which calls for the use of intracellular-acting antibiotics which are limited. Furthermore, the most effective regimens and treatment durations are still controversial (3). Therefore, significant research efforts have been carried out to seek and develop new and better therapies against *Brucella* infection.

Recently, there are numerous advances in immunology, genomics, proteomics, biochemistry as well as recombinant technology that have been utilized in the development of subunit vaccines through recombinant proteins (4). This kind of vaccine is able to reduce drawbacks of live attenuated vaccines including reversion to virulence, abortion in pregnant animals and infection to humans (5). Consequently, more studies have been reported on the protective efficiency of recombinant *Brucella* proteins as subunit vaccine against *Brucella* infection such as Outer membrane protein (Omp) 28, 50S ribosomal protein (L7/L12), Omp16, Omp19, lumazine synthase, etc… (6–9). Interestingly, combined subunit vaccine (CSV) using more than two recombinant proteins recently has been reported to confer higher potential immune response against *Brucella* infection than single subunit vaccine (10–13). Among them, recombinant proteins L7/L12 and Omps were considered as potential immunogens and demonstrated to induce strong protective effects against *Brucella* infection as well as others bacterial infections. Therefore, in this study, we evaluated the ability of a combination of four *B. abortus* recombinant proteins L7/L12, Omp16, Omp19, Omp28 as a CSV to induce immune response against *B. abortus* infection in RAW 264.7 cell line and BALB/c mouse models.

## Materials and Methods

### Bacterial Strains and Cell Culture

The smooth, virulent, wild-type *B. abortus* 544 biovar 1 strain (ATCC 23448) was cultured in Brucella broth at 37°C until stationary phase. *B. abortus* RB51 vaccine strain was used as positive control. *E. coli* DH5α was purchased from Invitrogen. *E. coli* cultures grown at 37°C in LB broth or agar supplemented with 100 μg/mL of ampicillin were used for expression of recombinant proteins. RAW 264.7 cells (ATCC, Rockville, USA) were grown at 37°C in 5% CO_2_ atmosphere in RPMI 1640 containing 10% (vol/vol) heat-inactivated FBS with or without antibiotics (100 U/mL penicillin and 100 μg/mL streptomycin) depending on the experiment.

### Recombinant Protein Expression and Purification

Preparation and expression of plasmids and purified recombinant proteins were obtained as described previously (13). Briefly, the fully coded sequences of the four *B. abortus* genes *Omp16, Omp19, Omp28*, and *rplL* were amplified using their respective primer pairs (Table 1). The individual amplified DNA fragments were cloned into a pCold-trigger factor (pCold-TF) vector. Recombinant proteins, namely rOmp16, rOmp19, rOmp28, and rL7/L12 were expressed in *E. coli* DH5α, and were purified using HisTALON gravity columns purification kit. The expression and immunoreactivity of these recombinant proteins were analyzed by SDS-PAGE and western blot assay, respectively.

**Table 1:** Primer sequences used for cloning *B. abortus* genes *Omp16, Omp19, Omp28*, and *rplL*.

**Gene**	**Forward primer**	**Reverse primer**	**Restriction enzyme (forward)**	**Restriction enzyme (reverse)**
*Omp16*	5′-CCC GGATCC^*a*^ ATGCGCCGTATCCAGTCGATT-3′	5′-ACC AAGCTT TTACCGTCCGGCCCCGTTGAG-3′	*Bam*HI	*Hind*III
*Omp19*	5′-AGCA GGATCC ATGGGAATTTCAAAAGCAAG-3′	5′-ATA CTGCAG TCAGCGCGACAGCG-3′	*Bam*HI	*Pst*I
*Omp28*	5′-GATC GGATCC AACACTCGTGCTAGCAATTTT-3′	5′-GATC AAGCTT TTACTTGATTTCAAAAACGAC-3′	*Bam*HI	*Hind*III
*rplL*	5′-AGC TCTAGA A TGGCTGATCTCGCAAAGATC-3′	5′-ATC CTGCAG CTTACTTGAGTTCAACCTTGGC-3′	*Xba*I	*Pst*I

a *Enzyme recognition sequences are underlined*.

### Mice Immunization and Bacterial Challenge

Twenty 12-week-old female BALB/c mice were distributed into four groups of five mice each. Each animal was intraperitoneally (IP) injected with a mixture of incomplete Freund's adjuvant (IFA) (Sigma-Aldrich, USA) and 100 μg of a combination of rOmp16, rOmp19, rOmp28, and rL7/L12 at a ratio of 1:1:1:1 in a total volume of 200 μL at weeks 0, 2, and 5. The other two groups were injected IP with phosphate buffered saline (PBS) or pCold-TF (100 μg) combined with IFA in a total volume of 200 μL at weeks 0, 2, and 5. Mice group used as positive control was IP immunized with 1 × 10^6^ CFUs of vaccine strain *B. abortus* RB51 in 100 μL PBS at day 0. Serum samples were collected via tail vein from all mice at week 7 after the first immunization to evaluate cytokine levels as well as IgG1 and IgG2a production. At week 7, mice were IP challenged with approximately 2 × 10^5^ CFUs of *B. abortus* 544 virulent strain in 100 μL PBS.

### Cytokine and Antibody Measurement From Serum Samples

The levels of IL-10, IFN-γ, TNF-α, IL-6, MCP-1 and IL-12p70 in serum samples were determined using a cytometric bead array kit (BD CBA Mouse Inflammation Kit, USA) and analyzed using a FACSCalibur flow cytometer.

The CSV-specific antibody titers IgG1 and IgG2a were measured using indirect ELISA. Briefly, the immunoassay 96-well plates were coated with 100 μL of a combination of four recombinant proteins (6 μg/mL) at a ratio of 1:1:1:1 in coating buffer (50 mM carbonate-bicarbonate coating buffer, pH 9.6) per well at 4°C, overnight. CSV-coated plates were washed, blocked, incubated with serial dilutions of sera, then with secondary antibody and results were analyzed following the previous method (13).

### Cytokine and NO Production in RAW 264.7 Cells

Overnight culture of RAW 264.7 cells at a concentration of 2 × 10^5^ cells per well in 96-well culture plates were pre-treated with lipopolysaccharide (LPS), pCold-TF or CSV for 4 h with PBS as control. The cells were washed with PBS, incubated in fresh medium (RPMI 1640 with 10% heat-inactivated FBS) and then infected with *B. abortus* at multiplicity of infection of 50. The cells were centrifuged at 150 × *g* for 10 min and incubated at 37°C in 5% CO_2_ for 1 h. Further, the cells were washed and added with fresh medium containing 50 μg/mL of gentamicin and treated with LPS, pCold-TF or CSV for 4, 24 and 48 h. At different time points (4, 24, and 48 h), 50 μL of cell culture supernatant from each well was collected to evaluate cytokine production using a cytometric bead array kit (BD CBA Mouse Inflammation Kit, USA) which was analyzed using a FACSCalibur flow cytometer. Another 50 μL of cell culture supernatant was collected to measure nitric oxide (NO) production using Griess reagent system (Promega, USA) according to the manufacturer's instruction.

### *In vivo* Bacterial Clearance Efficiency Assay

Two weeks after infection, all mice were sacrificed, and the spleens were collected, weighed, and homogenized in PBS. The homogenized spleens were serially diluted, plated on Brucella agar and incubated at 37°C for 3 days. The number of CFU per spleen was counted. Unit of protection was calculated as the mean log_10_ CFU of PBS group minus log_10_ CFU of vaccinated group.

### Statistical Analysis

The results for each of experiment are expressed as the mean ± standard deviation (SD). Data were analyzed by GraphPad InStat using unpaired, two-tailed Student's *t-*test. Results with *P* < 0.05 were considered statistically significant.

## Results

### Protein Purification and Immunoreactivity of Recombinant Proteins

**Figure 1:**
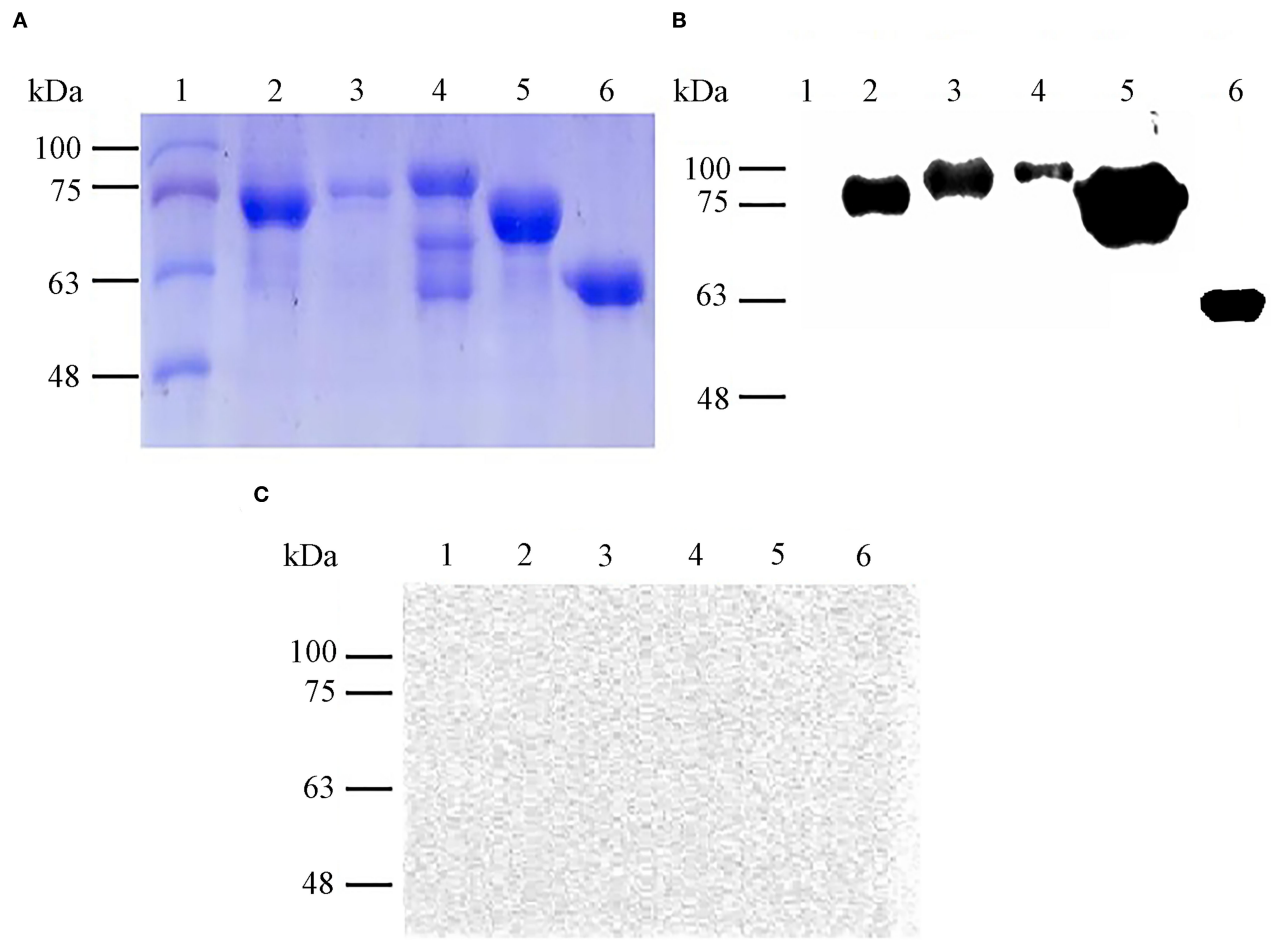
Expression and immunoreactivity of purified *B. abortus* recombinant proteins. Purified recombinant proteins were stained with Coomassie brilliant blue for SDS-PAGE analysis **(A)** and immunoreactivity of each antigen against *Brucella*-positive **(B)** or *Brucella*-negative mouse serum were determined by western blot assay **(C)**. Lane 1: molecular weight markers, lane 2: rOmp19 (77.60 kDa), lane 3: rOmp16 (78.23 kDa), lane 4: rOmp28 (86.55 kDa), lane 5: rL7/L12 (72.55 kDa), lane 6: pCold-TF (60.00 kDa).

By Coomassie brilliant blue staining, the approximate molecular masses of purified proteins rOmp19, rOmp16, rOmp28, rL7/L12, and pCold-TF were 77.60 kDa, 78.23 kDa, 86.55 kDa, 72.55 kDa, and 60.00 kDa, respectively (Figure 1A). On the other hand, the immunoreactivity of all purified proteins was measured by western blot assay. The results showed that these proteins reacted with *Brucella-*positive mouse serum and even with pCold-TF (Figure 1B) but all of them did not react with *Brucella-*negative serum (Figure 1C).

### Cytokine and NO Production in RAW 264.7 Cells Culture Supernatant

**Figure 2:**
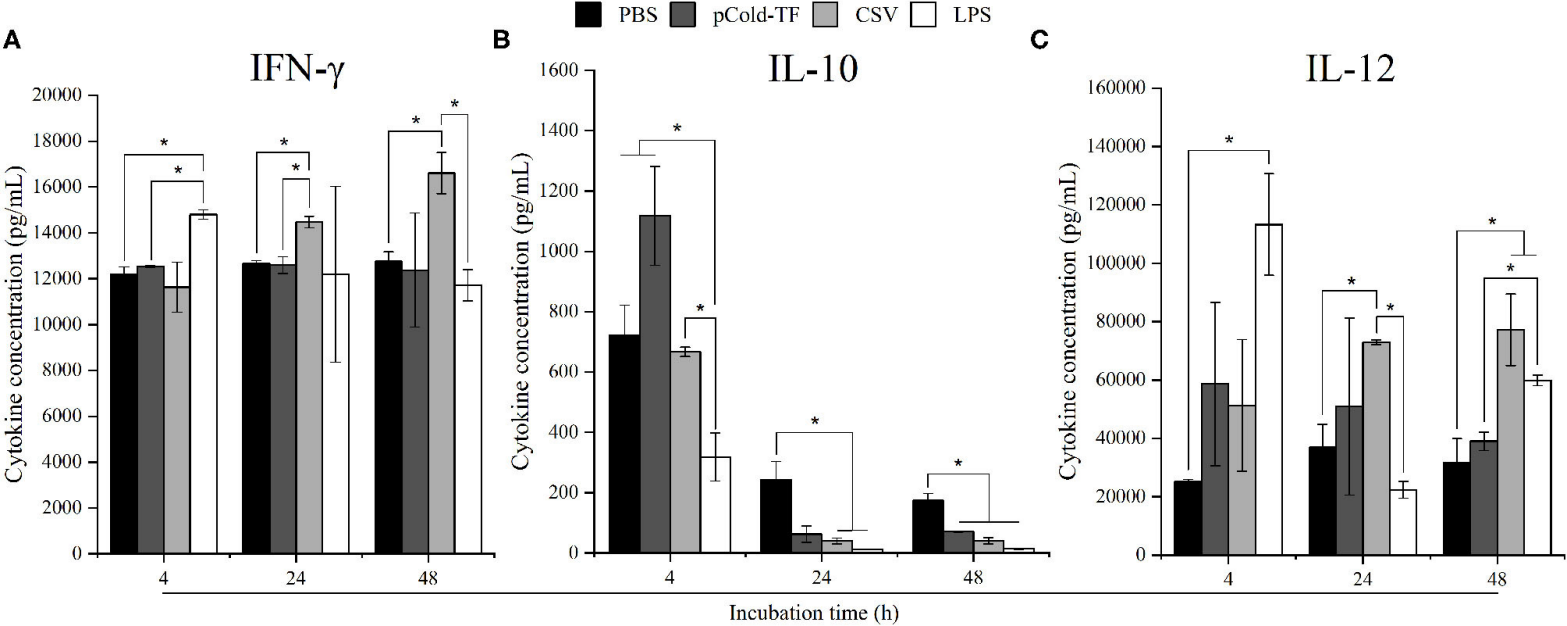
Cytokine concentration in RAW 264.7 cell culture supernatants. RAW 264.7 cells were pre-treated with LPS, pCold-TF or CSV for 4 h prior to infection. After infection, 50 μL of cell culture supernatant was collected and analyzed for cytokine production including IFN-γ **(A)**, IL-10 **(B)** and IL-12 **(C)**. The data are represented as the mean ± SD of duplicate samples from at least two independent experiments. Asterisks indicate statistically significant differences (*P < 0.05).

Macrophages are one of the first line of innate immune response against invading *Brucella* mediated by releasing an impressive panel of cytokines (14). Herein, cytokines production from cell culture supernatant was measured using flow cytometry assay. At the early stage of infection (4 h post infection), LPS-treated cells strongly produced 1.21-fold increase in IFN-γ and 4.51-fold increase in IL-12 but 2.27-fold decrease in IL-10, whereas no difference was observed in CSV-treated cells compared to PBS-treated cells. Interestingly, CSV-treated cells significantly induced 1.14-fold higher in IFN-γ and 1.98-fold higher in IL-12 production at 24 h post infection and continuously increased at 48 h, conversely, IL-10 level showed 6.14-fold and 4.26-fold decrease at 24 and 48 h post infection, compared to PBS-treated cells, respectively (Figure 2).

**Figure 3:**
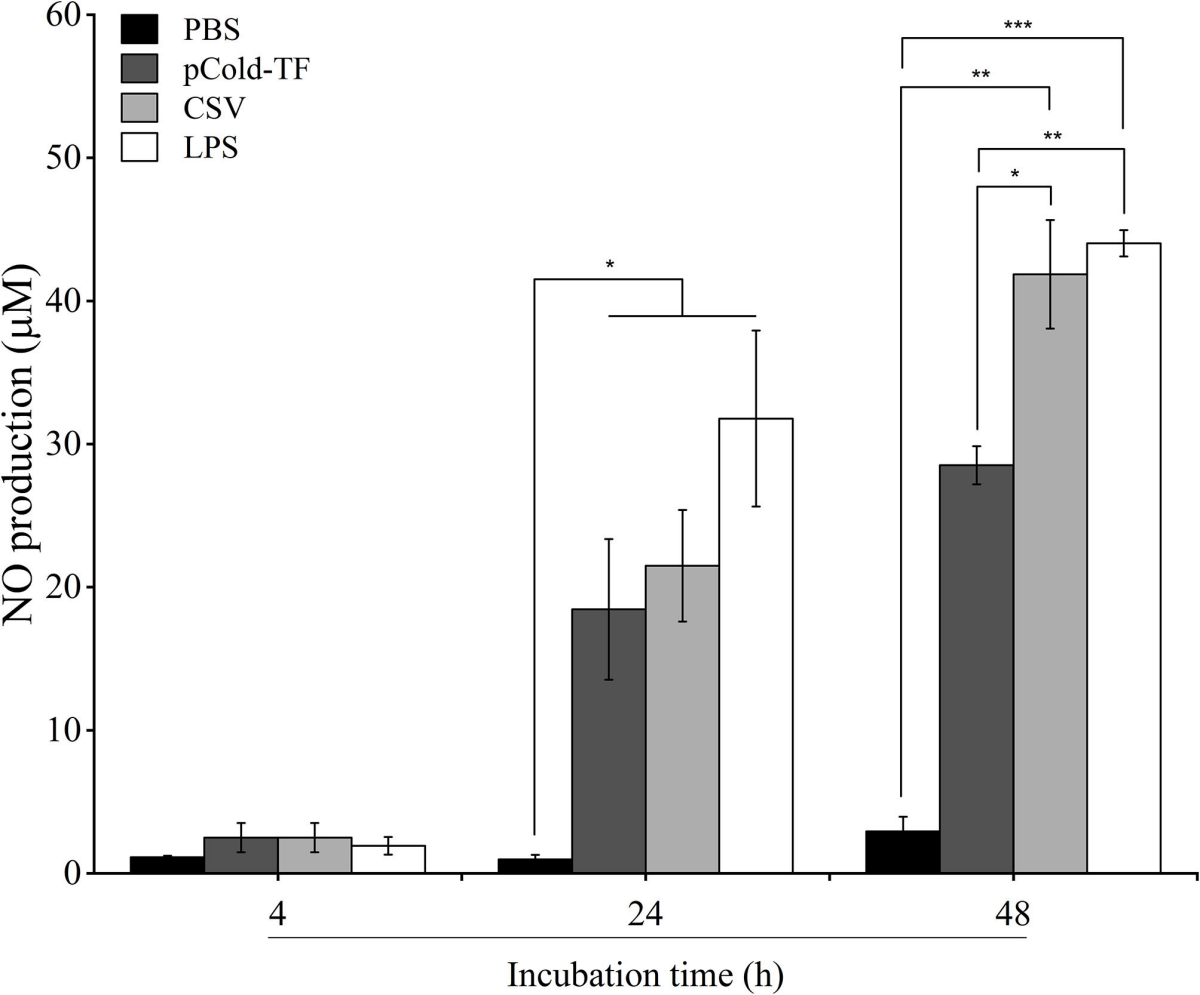
NO production in RAW 264.7 cell culture supernatants. RAW 264.7 cells were pre-treated with LPS, pCold-TF or CSV for 4 h prior infection. After infection, 50 μL of cell culture supernatant was collected and the accumulation of NO was analyzed using Griess reaction system. The data are represented as the mean ± SD of duplicate samples from at least two independent experiments. Asterisks indicate statistically significant differences (*P < 0.05, **P < 0.01, ***P < 0.001).

NO has been proven to control *Brucella* infection in macrophages (15). Therefore, in this study, NO production was measured to evaluate the antimicrobial mechanism employed by macrophages to combat *Brucella* infection. There was no significant difference in NO production at the early stage of infection (4 h post infection) in all treated and untreated cells. However, it was noteworthy at the late infection (24 and 48 h post infection) that all treated cells increased NO production compared to control cells whereas LPS-treated cells were the most predominant in NO production. Notably, at 48 h post infection NO level was observed to be continuously increased in CSV-treated cells and 1.47-fold higher than pCold-TF-treated cells (Figure 3).

### Cytokine Secretion Analysis in Serum Samples

**Figure 4:**
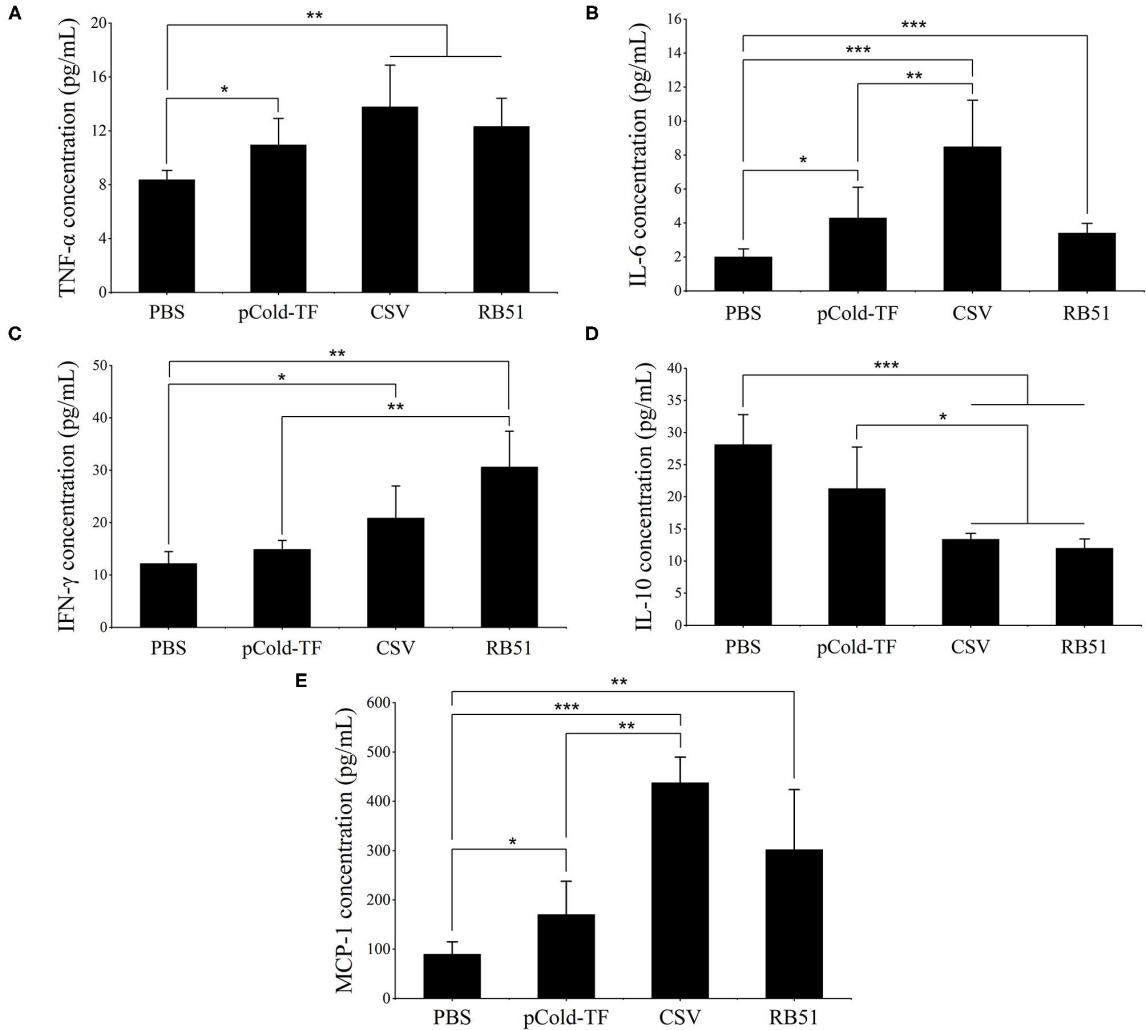
Cytokine concentration in the sera of immunized mice. Concentration of TNF-α **(A)**, IL-6 **(B)**, IFN-γ **(C)**, IL-10 **(D)** and MCP-1 **(E)** were analyzed using cytometric bead array. The data are represented as the mean ± SD of each group of five serum samples. Asterisks indicate statistically significant differences (*P < 0.05, **P < 0.01, ***P < 0.001).

At week 7 after the first immunization, sera were collected from all mice and cytokines levels were analyzed. CSV-immunized mice produced 1.65, 4.24, and 4.86-fold increases of pro-inflammatory cytokines TNF-α, IL-6 and MCP-1, respectively compared to PBS group. Moreover, CSV group displayed considerably increased IFN-γ production by 1.71-fold and decreased IL-10 production by 2.10-fold compared to PBS. Besides, CSV group elicited higher IFN-γ levels than IL-10 levels of approximately 1.56-fold. On the other hand, mice immunized with pCold-TF vector showed induced enhancement of TNF-α, IL-6 and MCP-1 levels compared to PBS group as well as induced IL-10 level that is ~1.43-fold higher than the IFN-γ level. Whereas, attenuated vaccine group RB51 remarkably induced highest IFN-γ level which is known to plays critical role in fighting against *Brucella* infection (Figure 4).

### Induction of Humoral Immunity by Eliciting Specific IgG1 and IgG2a Antibody in Immunized Mice

**Figure 5:**
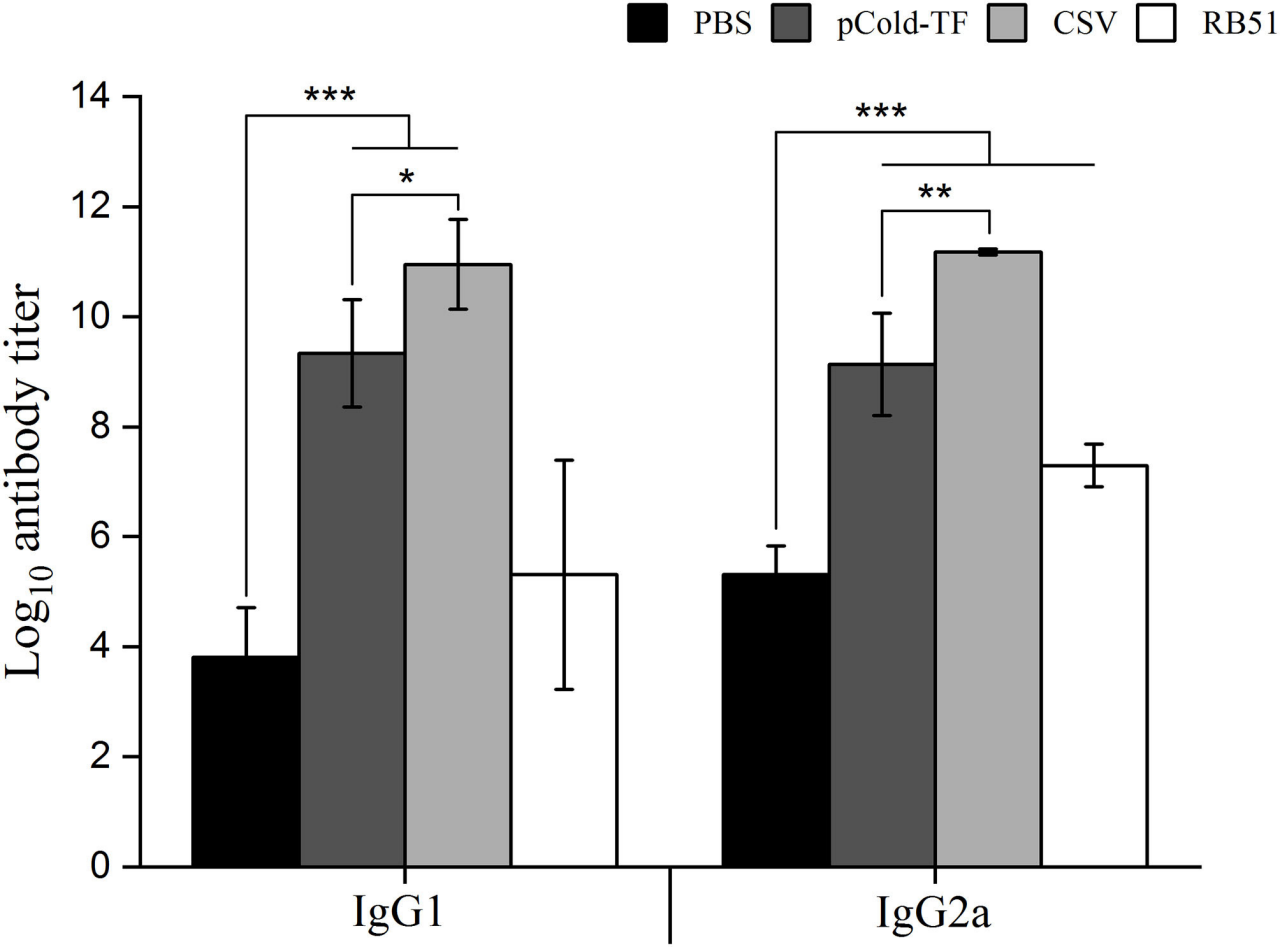
IgG1 and IgG2a antibodies production in the sera of mice immunized with different vaccines. Sera from CSV, pCold-TF, RB51, or PBS-immunized mice were collected at week 7 after the first immunization. CSV-specific IgG1 and IgG2a antibodies titer were determined by ELISA. The data are represented as the mean ± SD of each group of five serum samples. Statistically significant differences are indicated by an asterisk (*P < 0.05, **P < 0.01, ***P < 0.001).

Specific IgG1 and IgG2a antibodies produced by B lymphocytes, play substantial role in neutralization and opsonization which facilitate the phagocytosis of *Brucella* by some professional phagocytes. In this study, ELISA was utilized to measure the presence of CSV-specific IgG1 and IgG2a antibodies in the serum samples. The results showed that CSV group induced the highest IgG1 and IgG2a production in which the IgG2a/IgG1 ratio was 1.02. Besides, pCold-TF group displayed increased production of CSV-specific IgG1 and IgG2a compared to PBS group with IgG1/IgG2a ratio of approximately 1.02. On the other hand, RB51 group induced higher production of IgG2a than IgG1 by 1.37-fold (Figure 5).

### Protection Against *B. abortus* in Immunized Mice

**Figure 6:**
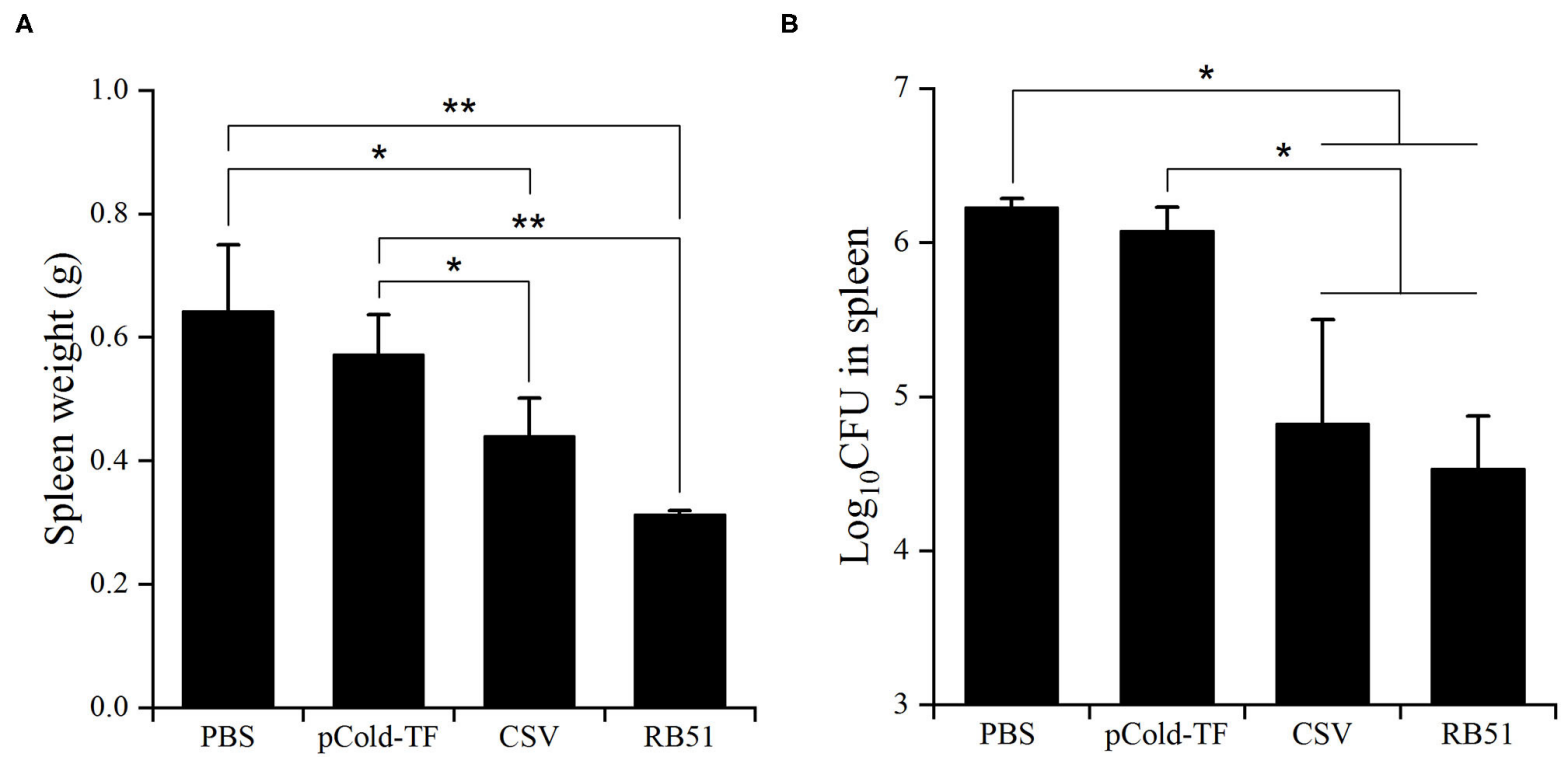
Protection against *B. abortus* in BALB/c mice immunized with different vaccines. The average total weight of each spleen was measured **(A)** and the number of CFUs in each spleen were counted **(B)**. The data are represented as the mean ± SD of five mice. Asterisks indicate statistically significant differences (*P < 0.05, **P < 0.01).

After three rounds of immunization, vaccinated and control mice were challenged by IP infection with *B. abortus*. Furthermore, CSV group conferred significant degree of protection with 1.41- and 1.26-log unit of protection compared to control mice receiving PBS and pCold-TF, respectively. RB51-immunized mice displayed highest degree of protection, approximately 1.70-log protection than PBS group. Mice immunized with pCold-TF vector exhibited induced 0.15-log protection compared to PBS group but not significant (Figure 6, Table 2).

**Table 2:** Protection against *B. abortus* in BALB/c mice immunized with different vaccines.

**Vaccine**	**Log_**10**_ CFU of bacteria in spleens (Mean ± SD)**	**Log protection**	***P*-value^**a**^**
PBS	6.23 ± 0.05		
pCold-TF	6.08 ± 0.15	0.15	
RB51	4.53 ± 0.34	1.70	*P* < 0.05
CSV	4.82 ± 0.68	1.41	*P* < 0.05

a *Significant different from PBS-immunized mice were estimated by Student's t-test*.

## Discussion

*Brucella* has the ability to survive, replicate and persist within professional and non-professional phagocytes. It is able to avoid degradation within phagolysosome fusions, reaching its safe haven-endoplasmic reticulum (ER). Once safely residing in ER, *Brucella* is able to evade bactericidal mechanisms within the professional phagocytes as well as pursuit of the humoral immunity (16). On the other side, the complex immune systems of mammals have evolved over vast periods of time when facing battles against pathogens. Among them, activated macrophages, dendritic cells, CD4^+^ and CD8^+^ T cells as well as various macrophages, dendritic cells, T helper (Th) 1-derived cytokines are predominant in protection against *Brucella* which possesses stealthy strategies to serve its intracellular nature (17).

Recently, growing numbers of studies have been widely carried out toward an ideal vaccine that would be able to prevent abortion in immunized host, bacterial infections in both immunized and non-immunized host and virulence reversion, and be able to promote long periods of protection with less doses and to be produced in large scale with low cost (18). Subunit vaccine using recombinant proteins was considered as an alternative preventive therapy which is able to fulfill the requirements of an ideal vaccine. Ribosomal protein L7/L12 functionally constitutes 50S ribosome encoded by *rplL* gene and plays an important role in controlling protein translational accuracy (19). Notably, it was demonstrated to be a known immunodominant antigen that stimulate strong immunity against *Brucella* infection (20, 21). The other agents of this study are Omps, essential to bacterial physiology and antibiotic resistance ability (22). *Brucella* Omps are classified based on their apparent molecular mass including group 1 (~88-94 kDa), group 2 (~41-43 kDa) and group 3 antigens (~30 kDa). Among them, Omp28 belongs to group 3. Two other Omps identified as lipoproteins are Omp16 and Omp19 (23, 24). In addition, immunogenicity of rL7/L12, rOmp16 and rOmp19 were evaluated effectively against *B. suis* and *B. melitensis* (20, 25, 26). Therefore, in the present study, a combination of four recombinant proteins L7/L12, Omp16, Omp19, and Omp28 was hypothesized to have the ability to activate strong immune responses against *Brucella* infection in RAW 264.7 cell line and BALB/c mouse models.

At the onset of host immunity activation against invading pathogens, cytokine MCP-1 regulates the migration and infiltration of macrophages, natural killer (NK) cells and T lymphocytes toward infection (27). In here, macrophages were described as sentinel of immunity, one of the first lines of innate immunity. They can display remarkable complexity of microbicidal functions including phagocytosis, phagolysosome fusion and production of reactive oxygen species (ROS), nitrite intermediates, antimicrobial peptides and degradative enzymes (28). The results in the present study showed that MCP-1 level in sera was observed to increase in CSV-immunized mice compared to PBS or pCold-TF group. In addition, production of two pro-inflammatory cytokines TNF-α and IL-6 was elevated in CSV group. These two pro-inflammatory cytokines are known as key effectors in mediating macrophages against *Brucella* infection. These cytokines promote phagolysosome fusion event as well as the production of killing effectors such as ROS, NO and lysosomal enzyme (29, 30). Furthermore, *in vitro* experiment showed significant enhancement of NO production in CSV-treated RAW 264.7 cells compared to PBS-treated cells during late stage of infection. These *in vitro* and *in vivo* experiments suggested that treatment with CSV could significantly initiate innate immunity, more particularly, activation of macrophages to elicit antimicrobial effectors that leads to restriction of an early infection.

In addition to playing a major role in innate immunity, macrophages can produce IL-12 to induce the activation of CD4^+^ Th1 cells (31). Although IL-12 production in sera in CSV-immunized mice was not detected, CSV-treated RAW 264.7 cells induced IL-12 production compared to PBS group. This result showed that macrophages played a role as functional bridge between innate and adaptive immunity. After naive T cells were activated to differentiate into Th1 by macrophages-derived IL-12, Th1 consequently produces IFN-γ. This Th1-derived IFN-γ is a crucial cytokine in immune responses against *Brucella* infection with several important functions (32). The present results showed that not only CSV-immunized mice but also CSV-treated RAW 264.7 cells displayed increased production of IFN-γ compared to control. This is consistent with the previous studies, IFN-γ concentration was obviously up-regulated when the host was immunized with rL7/L12 or rOmp19 in context of *B. suis* or *B. melitensis* infection (20, 25). In contrast, the anti-inflammatory cytokine IL-10 is a marked cytokine for Th2 activity. It inhibits activity of Th1, NK cells and macrophages leading to increase resistance of *Brucella* infection (33, 34). Thus, ratio of IFN-γ and IL-10 can provide an immune system profile reflecting predominance of either Th1 or Th2. In this study, production of IL-10 was decreased in CSV-immunized mice and -treated RAW 264.7 cells in sera and culture supernatant, respectively. Interestingly, at both *in vitro* and *in vivo* experiments, the concentration of IFN-γ was greater than the concentration of IL-10 in CSV-treated RAW 264.7 cells and immunized mice. These findings indicated that immunization with CSV induced Th1 T cells. Besides, humoral immune response mediated by antibodies assists in opsonisation of circulating *Brucella* in the blood of infected host. Elevated IgG1 and IgG2a antibodies production in sera in CSV-immunized mice were observed compared to PBS or pCold-TF-immunized mice. Collectively, alterations of cytokine and antibody profiles both *in vitro* and *in vivo* systems demonstrated that CSV effectively induced both Th1 and humoral immune responses.

Finally, the immunization with CSV conferred significant level of protection compared to PBS and pCold-TF groups. On the other hand, immunogenicity of pCold-TF was a notable result. It could react with *Brucella*-positive serum, induce production of TNF-α, IL-6 and MCP-1, whereas concentration of IFN-γ was < IL-10. This indicated that pCold-TF could induce Th2 immunity. Furthermore, this vector could elicit productions of IgG1 and IgG2a. The immunogenicity of this vector was primed by trigger factor component as reported by Cohen et al. (35) and Yang et al. (36). Although pCold-TF was able to elicit immune response, its protective effect against *Brucella* infection was not significant.

In conclusion, this study clearly indicated that immunization with a combination of four antigenic recombinant proteins rL7/L12, rOmp16, rOmp19, and rOmp28 could significantly induce Th1 immune response and humoral immunity, and provide superior protection effect against *Brucella* infection as compared to PBS and pCold-TF group. Although CSV could induce significantly protection effect, this study was conducted in a murine model which is not a natural host of *B. abortus*. Therefore, further investigations are needed to determine the practical efficacy of this vaccine using a bovine model which is considered as the natural host of this pathogen.

## Data Availability Statement

The original contributions generated for this study are included in the article/supplementary material, further inquiries can be directed to the corresponding author/s.

## Ethics Statement

The method of animal handling and sacrifice conducted in this experiment was in accordance with established federal guidelines and institutional policies approved by the Animal Ethical Committee of Chonbuk National University (Authorization Number CBNU-2018-101).

## Author Contributions

TH designed and performed the experiments, analyzed data and wrote original draft. TN, AR, and SV acquired data and revised manuscript. WM, HL, and JL contributed to conception and resources. SK contributed to conception, manuscript review and revision and project administration. All authors contributed to the article and approved the submitted version.

## Conflict of Interest

The authors declare that the research was conducted in the absence of any commercial or financial relationships that could be construed as a potential conflict of interest.
